# Evaluation of Morphology and Biochemical Parameters of Young Adults Using Heated Tobacco Products in Poland: A Case-Control Study

**DOI:** 10.3390/jcm14082734

**Published:** 2025-04-16

**Authors:** Małgorzata Znyk, Filip Raciborski, Dorota Kaleta

**Affiliations:** 1 Department of Hygiene and Epidemiology, Medical University of Lodz, Żeligowskiego 7/9, 90-752 Lodz, Poland; dorota.kaleta@umed.lodz.pl; 2 Department of Environmental Hazard Prevention, Allergology and Immunology, Warsaw Medical University, Banacha 1a, 02-091 Warsaw, Poland; filip.raciborski@wum.edu.pl

**Keywords:** biochemical markers, blood, health, heated tobacco product, IQOS, lipid profile, platelet count, smoking, tobacco, uric acid

## Abstract

**Background/Objectives:** Little is currently known of the impact of heated tobacco on health. The aim of this study is to evaluate the impact of heated tobacco use on selected health assessment parameters among people aged 18–30 to determine the effect on health status. **Methods:** A case-control study was conducted from April 2022 to February 2025. A total of 195 young, healthy adult residents of Lodz, Poland, took part. The participants were divided into three groups: IQOS (I-Quit-Ordinary-Smoking) smokers who had never smoked or who had quit smoking six months previously (*n* = 65); daily smokers who had smoked at least five cigarettes per day for at least one year and had not used any other smoking substitutes for at least one year (*n* = 65); and people who have never smoked or used tobacco products (*n* = 65). Blood samples from 37 IQOS users (57%), 28 traditional cigarette smokers (43%), and 45 non-smokers (69%) were submitted for laboratory analysis. The tested parameters were determined in the diagnostic laboratory of the Bonifratów Hospital in Lodz. **Results:** No significant differences (*p* > 0.05) were found between the groups with regard to blood count (white blood cell count (WBC), red blood cell count (RBC), lymphocytes, monocyte number (MONO), hemoglobin concentration (HGB)), biochemical biomarkers (C-reactive protein (CRP), fibrinogen, apolipoprotein A1 (apo A1), apolipoprotein B (apo B), glucose), or lipid profile (total cholesterol, triglycerides (TG), high-density lipoprotein (HDL), low-density lipoprotein (LDL)). The cigarette smokers demonstrated significantly higher uric acid levels compared to the IQOS users and non-smokers: 5.22 vs. 4.77 vs. 4.40 mg/dL (*p* < 0.01). The IQOS users demonstrated significantly higher platelet count levels compared to cigarette smokers and non-smokers: 290.27 vs. 267.14 vs. 256.33 × 10^3^/ μL (*p* < 0.05). Among the IQOS users (*n* = 37), the level of glucose (ρ = −0.47; *p* = 0.01), WBC (ρ = −0.36; *p* = 0.03), lymphocytes (ρ = −0.38; *p* = 0.02), and uric acid (ρ = −0.34; *p* = 0.04) was negatively correlated with the daily number of heated tobacco sticks. The HDL level was positively correlated (ρ = 0.39; *p* = 0.02) with the daily number of heated tobacco sticks. **Conclusions:** Further cohort studies assessing the health status of young users of heated nicotine products and prospective analyses are necessary.

## 1. Introduction

Heated IQOS (I-Quit-Ordinary-Smoking) tobacco products are being promoted as alternatives to traditional cigarettes, with the attraction that their use is less harmful than smoking [1,2]. IQOS *heat-not-burn* tobacco products were first introduced in 2014 as a pilot project in Italy and Japan by PMI (Philip Morris International) [2]. Over the next few years, they appeared in Canada, England, and the United States [3,4,5], and they were introduced to the Polish market in 2017 [6]. Such devices are currently sold in over 50 countries [7], including at least 20 countries in Europe [8]. The IQOS tobacco heating system works at lower temperatures (240–350 °C) than traditional cigarettes (>600 °C) [1], allowing the aerosol to be produced without burning tobacco [9]. IQOS is the most popular heated tobacco product on the market next to Ploom TECH and glo [1].

The use of heated tobacco products (HTP) has increased in recent years [10,11,12], with the incidence rising from 0.2% in 2015 to 11.3% in 2019 in Japan [11]. In Poland, daily use rose tenfold from 0.4% of respondents in 2019 to 4% in 2022 [12,13]. Currently, approximately 1.5% of the global population report regularly using HTPs, and 5% have tried them [10]. In addition, 1.3% of the population in Europe were current users in 2020, with 0.7% using them daily [14]. Reviews also confirm increasing use of heated tobacco products in American, Pacific, and European countries [10,15].

The manufacturers state the purpose of the IQOS system is to minimize exposure to the hazardous substances present in cigarette smoke and thus reduce the likelihood of developing tobacco-related diseases. In doing so, this should provide a safer alternative for current smokers [16,17].

Independent and industry-specific studies have both shown that IQOS sticks contained 70–80% of the concentration of nicotine found in traditional cigarettes [18,19,20]. Recent research has also found that IQOS emits significantly lower levels of tar, carbonyls, VOCs (volatile organic compounds), CO (carbon monoxide), nitrosamines, and free radicals compared to traditional cigarettes. However, while HTP use may reduce the risk of developing tobacco-related diseases, it may still constitute a potential threat to human health [21,22,23]. Experimental studies on cells and animals have shown that aerosol from heated tobacco products has lower toxicity compared to cigarette smoke [1].

Unfortunately, no studies have yet fully assessed the use and potential health effects of HTPs, especially among young people [24]. Furthermore, as IQOS use may serve as an entryway to nicotine addiction among non-smokers, rather than as a substitute for tobacco smoking [25], there is a need to better understand their use and potential negative impact. This is particularly important considering the great interest in these products demonstrated by both smokers and non-smokers [26]. 

Following aggressive marketing by the tobacco industry, the number of HTP users has increased rapidly in recent years, especially those aged 18–30 years [24], who may be attracted by the technological design [27]. Alternatively, the popularity of HTPs among younger people may be due to their desire to follow a current trend or use a “safe cigarette”. Data suggest that IQOS may encourage young adults and adolescents to start using tobacco [28,29]. The use of IQOS has been found to encourage tobacco use with IQOS among young non-smokers and may also increase the use of IQOS, along with other tobacco products [30]. An analysis performed in various countries found the willingness to try IQOS to be universally lower than in the case of e-cigarettes but higher than compared to traditional cigarettes [31]. As such, there is a need to determine the characteristics of potential users, the level of use of the products, and their potential effects on health.

Only limited data are currently available on the impact of heated tobacco on health, with only single independent studies being available, and the existing studies are mainly restricted to examining the short-term health effects [1,32,33,34,35,36]. Nevertheless, it has been shown that the use of heated tobacco products can cause inflammation, increase oxidative stress, and elevate the risk of respiratory infections [32,37]; it can also impair the diastolic and systolic function of the myocardium in the acute phase, similar to conventional cigarette smoking [5,38].

Long-term HTP stimulation has been found to affect the differentiation and keratinization of gingival epithelial cells and may be a risk factor for oral mucosa diseases [39]. However, exposure to the aerosol from HTPs containing nicotine has a less harmful effect on the periodontium compared to traditional cigarettes [40].

In addition, research indicates that various forms of nicotine and tobacco products (including e-cigarettes) increase the risk of periodontitis, stomatitis, and oral cancer [41,42,43]. Short-term use of heated tobacco products in young adults has an immediate effect on vascular function, through the formation of platelet clots and increased arterial stiffness, these being risk factors for the development of atherosclerosis [35]. Other international studies have shown use of HTPs to be associated with increases in heart rate, pulse rate, blood pressure, and arterial stiffness [5,33,35,44,45,46,47], similar to traditional cigarettes, linking these products to increased cardiovascular risk [5,33]. Another study found chronic HTP use to be associated with a potential risk of cardiac fibrosis [48]. Users of heated tobacco products were also more likely to develop prediabetes and diabetes than non-smokers [49].

A study of the acute pulmonary effects of HTP aerosol exposure found IQOS to affect exhaled CO (carbon monoxide), SaO_2_% (oxygen saturation), and airway function immediately after use. While these changes were rather small, their presence should raise concerns about the long-term safety of the product [50]. Other studies report disturbances in the cytokine profile among people who regularly use heated tobacco [51], indicating that users are at increased risk of respiratory diseases; for example, increased levels of IL-8 (interleukin 8) have been shown in HTP users [51].

IQOS use increases susceptibility to infection-induced asthma attacks or respiratory infections. Exposure to IQOS, similar to exposure to traditional cigarettes and e-cigarettes, leads to mitochondrial dysfunction, which can further lead to airway remodeling, respiratory diseases, and lung cancer [27].

Two case reports have described the occurrence of acute eosinophilic pneumonia associated with HTP use in younger men who were non-smokers: a 20-year-old who had initiated HTP use six months previously and a 16-year-old two weeks earlier (20 HTP cigarettes per day and doubling of intake in the previous two weeks) [52,53,54].

A 40-year-old IQOS user was diagnosed with an IQOS-related lung injury, characterized by a unique clinical picture and a favorable response to smoking cessation [55]. In a three-year study, smokers with chronic obstructive pulmonary disease (COPD) who switched to HTP or stopped smoking showed consistent improvements in lung function, quality of life, exercise tolerance, and frequency of disease exacerbations compared with COPD patients who continued smoking [56]. In addition, people who used HTP for more than three years were found to be at increased risk of developing metabolic syndrome. This risk was higher than in those who smoked traditional cigarettes [57].

Due to the short time that HTPs have been on the market, their short- and long-term health effects still remain unclear. Hence, our study is the first to assess the impact of HTP use on blood biomarkers among young people in Poland. Changes in blood parameter values, i.e., outside the reference range, may indicate a harmful effect on health and, in the context of long-term use, lead to chronic diseases, including cardiovascular and respiratory diseases.

The aim of this study was to evaluate the impact of heated tobacco use on health status among young people, aged 18–30, using selected health assessment parameters. More specifically, it examines the effect of heated tobacco use on selected blood morphological parameters (red blood cell count, white blood cell count, monocyte number, platelet count, hemoglobin and C-reactive protein concentrations, uric acid, fibrinogen), lipid parameters (triglycerides, low-density lipoprotein, high-density lipoprotein), apolipoprotein A1, apolipoprotein B, and glucose levels among young people; it also compares the results between IQOS users, traditional cigarette smokers, and non-smokers.

## 2. Materials and Methods

### 2.1. Study Design and Participants

A case-control study was conducted from April 2022 to February 2025. In total, 195 young, healthy adult residents of Lodz, Poland, took part. To avoid bias, 65 participants were assigned to each group. The participants were divided into three groups: (1) IQOS smokers who had never smoked or who had quit smoking six months previously (*n* = 65); (2) daily smokers who had smoked at least five cigarettes per day for at least one year and had not used any other smoking substitutes for at least one year (*n* = 65) (this period was chosen to differentiate the health effects associated with exposure to heated tobacco); and (3) people who have never smoked or used tobacco products (*n* = 65). A regular smoker was assumed to be one who has smoked at least five cigarettes a day for at least a year. IQOS smokers were those who had smoked at least five sticks of heated tobacco daily for a minimum of six months before the start of the study and were not consuming other tobacco products.

The respondents were recruited from universities, colleges, and post-secondary schools in Lodz. Recruitment took place via the Internet, social media, and IQOS sales points. Individuals enrolled in the study by phone or via the study email. After a brief interview about smoking status and health, those meeting the inclusion criteria were informed about the study objectives and signed their informed consent to participate. The participants were given a paper questionnaire to complete and underwent anthropometric measurements. The data were digitalized for statistical analysis.

The National Science Center funded the study, research project no. 2021/41/N/NZ7/00020.

### 2.2. Exclusion and Inclusion Criteria

Inclusion criteria for heated tobacco users: 18–30 years of age, smoking only IQOS during recruitment, and smoking at least five sticks of heated tobacco product per day for at least six months before taking part. Inclusion criteria for daily smokers: age 18–30 years, smoking five or more cigarettes per day for at least a year.

Exclusion criteria for IQOS smokers, daily smokers, and non-smokers: co-existing diseases, especially cardiovascular diseases, chronic respiratory diseases, endocrinological diseases, different types of cancer, diabetes, neurological disease, hepatitis B and hepatitis C, liver cirrhosis, renal failure, rheumatoid arthritis, arteriosclerosis; history of surgery during the three months preceding the examination; regularly using psychoactive substances; or alcohol addiction. A control group was recruited to match the demographic structure of the heated tobacco smoking group.

### 2.3. Ethics

This study was given permission from the Bioethics Committee at the Medical University of Lodz (number RNN/290/21/KE of 14 December 2021) and was performed in accordance with the Declaration of Helsinki. All the participants provided written informed and voluntary consent.

### 2.4. Study Variables

All the participants read the study protocol and gave their consent to participate. They were then administered a standardized questionnaire, which had been adapted to the study from the GATS (Global Adults Tobacco Survey) questionnaire [58]. The questions concerned socio-demographic characteristics (sex, age, level of education, marital status, place of residence, year and field of study, type of university), smoking status (IQOS smoking, tobacco smoking, e-cigarette smoking), and health status and previous diseases in childhood and early adulthood. The users of HTPs were asked about their frequency of use during the day, the choice of heated tobacco stick, motivation to use IQOS, the circumstances and age at first use of IQOS, perception of the harmfulness of IQOS, and the addictive potential of IQOS.

Smokers of traditional cigarettes were asked about the frequency of smoking, motivation to smoke, the number and type of cigarettes smoked per day, the circumstances and age at starting smoking, perception of the harmfulness of smoking, and attempts to quit smoking.

The respondents were asked to subjectively assess their health condition as (1) very good, (2) good, (3) neither good nor bad, or (4) unsatisfactory. Additionally, the respondents indicated whether their health status was (1) better, (2) comparable, or (3) worse than the health status of their peers. The next question concerned the occurrence of symptoms in the last month, such as chest pain after exercise, joint pain, spine pain (back, low back), swelling (swelling of the feet), varicose veins of the legs, constipation, headache, insomnia, depression, and toothache. The participants reported the number of steps taken daily, calculated by electronic pedometers.

Anthropometric measurements were taken (height, weight, and waist circumference), and these were used to calculate BMI (body mass index) and WHR (waist-to-hip ratio). BMI (kg/m^2^) was calculated according to the following formula: body weight (kg) divided by the square of height (m^2^) [59]. Subjects with a BMI ≥30 kg/m^2^ were obese, those with a BMI between 25 and 29.9 kg/m^2^ were overweight, and those with a BMI 18.5 to 24.9 kg/m^2^ had normal body weight. The WHR was calculated by dividing the waist circumference (cm) by the hip circumference (cm); values > 94 cm in men and >80 cm in women indicated abdominal obesity, with a significant risk of metabolic complications [60].

In the second stage, the subjects were referred to a collection point for a laboratory blood test.

### 2.5. Measured Parameters

Blood was collected using a closed vacuum system in accordance with the rules for collecting biological material for laboratory tests [61]. Blood for testing (20 mL) was collected in the morning (between 7:00 a.m. and 10:00 a.m.), after sleeping, on an empty stomach (12 h after the last meal).

The following parameters were analyzed:

1. Blood morphological parameters: white blood cell count (WBC), red blood cell count (RBC), platelet count (PLT), monocyte number (MONO), hemoglobin concentration (HGB), fibrinogen (FBG), C-reactive protein (CRP), and uric acid (UA).
2. Lipid parameters and glucose level: triglycerides, LDL (low-density lipoprotein), HDL (high-density lipoprotein), total cholesterol, apo A1 (apolipoprotein A1), apo B (apolipoprotein B), and glucose level.

All the procedures were performed at the Medical University of Lodz and at the collection point of the Bonifratrów Hospital in Lodz under contract. The tested parameters were determined in the diagnostic laboratory of the Bonifratów Hospital in Lodz.

Diagnostic procedures were performed by qualified laboratory diagnosticians, as specified by the manufacturers of the diagnostic tests. Hematological tests were performed on an HN-550 analyzer from SYSMEX (SYSMEX EUROPA SE, Norderstedt, Germany). RBC was determined using the impedance method with hydrodynamic focusing, and WBC by flow cytometry. MONO was determined by fluorescence flow cytometry, PLT by impedance with hydrodynamic focusing, and HGB by SLS without the use of cyanides. Coagulation tests were performed on a WERFEN ACLTOP350 analyzer (WERFEN, Warsaw, Poland). Fibrinogen was determined using the Claus method. Biochemical tests were performed on a COBAS PURE analyzer by ROCHE (ROCHE, Warsaw, Poland). Parameters such as glucose, CRP, UA, CHOL, TG, HDL, and LDL were determined by photometry. Reference values of the tested parameters are provided in the Supplementary Materials (Table S1 Supplementary Materials).

No repeatability control was performed at the laboratory, and reproducibility control was carried out on control material from Sysmex (hematology), Roche (biochemistry), and Werfen (coagulology). Regarding potential inter-laboratory variability, the Analytical Laboratory is audited by the Central Center for Quality Research in Laboratory Diagnostics in Lodz (COBJwDL). The control material received by the Analytical Laboratory from the Center is subjected to specific tests; the obtained results are then processed statistically and compared with the results of other control participants working on the same analyzers, using the same methods and reagents. Studies with estimated errors are later sent to individual participants in the external control.

### 2.6. Statistical Analyses

The statistical analysis was performed using IBM SPSS 29.0.0. (Armonk, NY, USA, IBM Corp.) and STATISTICA 13.3 (Krakow, Poland, StatSoft Poland Inc.). For quantitative features, the range, mean values (arithmetic means and medians), and measures of internal diversity (standard deviations) were calculated.

The normality of distributions was tested using the Shapiro–Wilk test and histograms. The three groups of participants were compared quantitatively using the parametric one-way ANOVA for normally distributed variables, and the non-parametric Kruskal–Wallis test for non-normally distributed variables.

If the differences were significant, additional post hoc tests were performed to distinguish different groups. Pairwise comparison and the Bonferroni test were used.

Effect sizes for ANOVA were calculated between groups using eta-squared (η^2^) (<0.01 no effects; 0.01–0.06 small effect; 0.061–0.14 medium effect; >0.14 large effect).

Spearman’s rank correlation was used to analyze the correlation between the level of biomarkers in the blood and the daily number of heated tobacco sticks (strength of correlation: ρ < 0.2 no linear relationship; ρ = 0.2–0.4 weak relationship; ρ = 0.4–0.7 moderate relationship; ρ = 0.7–0.9 quite strong relationship; ρ > 0.9 very strong relationship). The correlation coefficient is provided with a sign to indicate a negative or positive correlation, respectively: a negative correlation represents an increase in one variable and a decrease in the other, while a positive correlation reflects an increase or a decrease in both variables together.

This study took into account potential confounding factors, such as age, sex, physical activity, and BMI, which could have influenced the blood parameters. Logistic regression was performed for platelet count, glucose, and uric acid level, adjusting for the confounding factors.

Statistical significance was demonstrated at *p* < 0.05. The details of the study methodology and preliminary results have been previously published [62].

## 3. Results

### 3.1. Characteristics of the Study Participants

In total, 195 people were recruited for the study: 65 IQOS smokers, 65 traditional cigarette smokers, and 65 never-smokers. The mean ages were 21.23 ± 2.02 years for the IQOS users, 21.43 ± 2.43 years for the traditional cigarette smokers, and 20.95 ± 1.81 years for the non-smokers. The majority of respondents were women (69.2% of IQOS users, 58.5% of traditional cigarette smokers, and 86.2% of non-smokers), and most were single (64.6% of IQOS users, 70.8% of traditional cigarette smokers, and 83.1% of non-smokers).

Most participants had completed post-secondary education (55.4% of IQOS users, 40.0% of traditional cigarette smokers, and 52.3% of non-smokers). Approximately 90.8% of IQOS users, 76.9% of smokers of traditional cigarettes, and 100% of non-smokers were students. All of the respondents were students of public universities. The majority of students were in years 1 to 3 of their studies (86.4% of IQOS users, 90.0% of smokers of traditional cigarettes, and 98.5% of non-smokers), and most were studying medicine (89.8% vs. 70.0% vs. 98.5%, respectively). Most participants lived in cities with over 200,000 inhabitants (67.7% of IQOS users, 55.4% of smokers of traditional cigarettes, and 47.7% of non-smokers). The socio-demographic data are presented in more detail in Table 1.

Young IQOS users most often used five heated inserts a day (37.0%), and most had been using IQOS for two years (30.7%). All the subjects used nicotine-containing cartridges (Table S2 Supplementary Material).

### 3.2. Subjective Health Assessment and Anthropometric Measurements

The subjective assessment of the health condition of the respondents is presented in Table 2. The majority (73.9%) of IQOS users rated their health condition as good. Almost half of the non-smokers (46.1%) and smokers of traditional cigarettes (50.8%) also rated their health as good. In addition, 84.6% of IQOS users, 60.0% of smokers of traditional cigarettes, and 67.7% of non-smokers indicated that their health condition was comparable to that of their peers.

The most common symptoms occurring in the previous month were headache, back pain, and insomnia (Table 2). Every third IQOS user reported health symptoms caused by using heated tobacco products; the most common were dizziness (13.8%), headaches (12.3%), and breathing difficulties (10.8%) (Table S2 Supplementary Material).

Among the IQOS users, 24.6% were overweight and 6.2% were obese. Among the respondents who smoked traditional cigarettes, 35.4% were overweight and 7.7% were obese. In the non-smokers, only 9.3% were overweight and 1.5% were obese. Abdominal obesity was diagnosed in every third non-smoker, 18.5% of IQOS users, and 23.1% of those who smoked traditional cigarettes (Table 2).

### 3.3. Blood Count, Biochemical Biomarkers, and Lipid Profile

Blood samples from 37 IQOS users (57%), 28 traditional cigarette smokers (43%), and 45 non-smokers (69%) were submitted for laboratory analysis. A comparison of the blood count parameters and biochemical biomarkers between the three groups is presented in Table 3. Reference values of the determined blood parameters and biomarkers are presented in Table S1 (Supplementary Materials). Table S3 (Supplementary Materials) presents the laboratory test results in the analyzed groups.

No significant differences (*p* > 0.05) were found between the groups with regard to blood count (WBC, RBC, lymphocytes, MONO, HGB), biochemical biomarkers (CRP, fibrinogen, apo A1, apo B, glucose), or lipid profile (total cholesterol, TG, HDL, LDL). The results are presented in Table 3.

The cigarette smokers demonstrated significantly higher uric acid levels compared to the IQOS users and non-smokers: 5.22 vs. 4.77 vs. 4.40 mg/dL (*p* < 0.01). The IQOS users demonstrated significantly higher platelet count levels compared to the cigarette smokers and non-smokers: 290.27 vs. 267.14 vs. 256.33 × 10^3^/ μL (*p* < 0.05).

A preliminary analysis showed that other blood parameters were not statistically significant; therefore, they are not discussed in detail in the Results section.

The effect sizes for the ANOVA analysis of blood parameters are presented in the Supplementary Materials (Tables S4 and S5). For PLT, eta-squared was 0.058; this is classed as a weak effect (η^2^ 0.01–0.06).

The value of the correlation coefficient in most analyses is negative, i.e., the level of the tested blood parameters decreases as the number of nicotine-heated tobacco sticks increases. Only single index values indicate a positive correlation but with a weak correlation.

Among the IQOS users (*n* = 37), the level of glucose (ρ = −0.47; *p* = 0.01), WBC (ρ = −0.36; *p* = 0.03), lymphocytes (ρ = −0.38; *p* = 0.02), and uric acid (ρ = −0.34; *p* = 0.04) was negatively correlated with the daily number of heated tobacco sticks. The HDL level was positively correlated (ρ = 0.39; *p* = 0.02) with the daily number of heated tobacco sticks (Table 4).

A logistic regression model was created for platelets, taking into account confounding factors, such as age, sex, BMI, and physical activity. The results are given in the Supplementary Materials (Table S6).

To verify the results, it was intended to create regression models including variables such as sex, age, BMI, and physical activity (number of steps). However, due to the low effective sample size and the different uric acid norms for women and men, it was not possible to create regression models for uric acid or glucose.

## 4. Discussion

This is the first study to investigate the effect of IQOS use on blood morphology and biochemical parameters in young people. The limited data available to date indicate that short-term use of HTP in healthy young adults has an immediate adverse effect on vascular function, causing increased arterial stiffness and the formation of plaque clots, which are known risk factors for the development of atherosclerosis [35]. Other studies report minor airway obstruction and resistance, as well as nicotine-related cardiovascular burden following the use of heated tobacco products [33].

The majority of our study participants in the three analyzed groups subjectively assessed their health as good. More than half of the participants in the non-smoking group and the group smoking traditional cigarettes believed that their health condition was comparable to that of their peers. Interestingly, as many as 85% of IQOS users believed that their health was comparable to that of their peers.

The highest incidence of overweight participants was recorded in the traditional cigarette group, with slightly lower rates recorded in the IQOS group. Our results confirm previous findings that smoking is causally associated with higher BMI, hip circumference, waist circumference, and waist-to-hip ratio [63]. Significant interactions have also been found between smoking, abdominal obesity, and the risk of cardiovascular diseases [64]. It has been proposed that smoking reduces fat storage capacity in the lower body, which, in turn, influences cardiovascular risk [65]. Smoking status has been found to be negatively associated with being a healthy weight and underweight and positively associated with being obese and overweight [66].

Blood tests can be a sensitive indicator of many diseases [67]. In the present study, no significant differences with regard to morphological parameters were observed between the IQOS users, cigarette smokers, and non-smokers. However, there is a need to conduct further research in this area among young people.

No significant differences in blood parameters (WBC, RBC, MONO, HGB), biochemical biomarkers (CRP, fibrinogen, apo A1, apo B, glucose), and lipid profile (total cholesterol, TG, HDL, LDL) were found between the study groups. However, other studies indicate that some biomarkers, such as high-sensitivity C-reactive protein, white blood cell count, apolipoprotein A1, and fibrinogen, correlated with smoking in a dose-dependent manner and were modifiable after smoking cessation [68,69]. Smokers switching from cigarettes to heated tobacco products demonstrated improvements in apolipoprotein A1 compared with the group smoking traditional cigarettes [68,69].

Only significantly higher platelet counts were demonstrated in the IQOS users compared to the non-smokers and smokers. Platelets play a key role in maintaining blood homeostasis and preserving the integrity of the vascular system.

There are conflicting results for PLT between the non-smokers and smokers [70]. Research shows that smoking increases PLT activity and PLT number [71]. Passive smokers had higher levels of platelets count [72]. Other studies have shown that mean PLT counts were significantly lower in smokers versus in non-smokers [73]. Recent studies have shown that PLT thrombus formation significantly increased following HTP exposure compared to no exposure [35]. The increase in platelets may also be related to lifestyle factors, such as diet and physical activity, or to measurement errors.

However, it should be remembered that these are preliminary results, and more advanced analyses are planned. Human studies conducted so far have been limited to the short-term health effects of IQOS use, and they have tended to be based on laboratory measurements taken after using heated tobacco products [1,32,33,34,36,37]. Previous studies have failed to identify any differences in the risk of serious or adverse events between heated tobacco product users and traditional smokers, or among people with short-term abstinence from tobacco [36].

Similarly, no significant differences in WBC counts were noted previously between smokers and non-smokers, or within the same group before and after exposure to cigarette smoke [74]. However, other studies have found cigarette smoking to have serious negative effects on hematological parameters, such as WBC count, hemoglobin level, mean red blood cell volume, mean RBC hemoglobin concentration, RBC count, and hematocrit [75]. Smokers have been found to have an increased total number of white blood cells [75] and leukocytes, a higher RBC count, and increased RBC parameters [68,70,75,76,77]. Smokers have also been found to have significantly higher levels of hemoglobin and mean red blood cell volume [75]. These changes may be associated with an increased risk of polycythemia vera, atherosclerosis, chronic obstructive pulmonary disease, and cardiovascular diseases [75].

After cessation of smoking, decreases have been noted in lymphocyte, neutrophil, and white blood cell counts [77]. A significant decrease in neutrophil counts was noted in smokers over three days when switching to electrically heated cigarettes or quitting smoking for a short time [78]. Abstinence leads to a rapid decline in WBC, caused by changes in the number of neutrophils [79], as well as lower RBC, hemoglobin, and hematocrit levels [79].

Other studies have shown increased monocyte levels in smokers [68], as well as some small increases in basophil and eosinophil counts in traditional cigarette and e-cigarette smokers compared with non-smokers. Eosinophilic granulocytes have been associated with respiratory disease. Some studies have noted similar levels of blood eosinophils in non-smokers and smokers [68].

Furthermore, smoking has been found to affect serum lipid levels and high cholesterol levels, thus increasing cardiovascular risk in smokers [80,81]. Smokers have higher levels of triglycerides (TG), low-density lipoprotein cholesterol (LDL-C), total cholesterol (TC), and lower levels of high-density lipoprotein cholesterol (HDL-C), which are associated with an elevated risk of cardiovascular disease [80]. It has been confirmed that quitting smoking resulted in an improved lipid profile, manifested as a decrease in LDL and an increase in HDL [82], and that switching smokers to HTP resulted in a significant improvement in biomarkers such as HDL-C and WBC [68,83]. The HDL level, triglyceride level, and WBC count were shown to be significantly different in an HTP group compared to a traditional cigarette group [84]. In another study, HTP users demonstrated higher HDL-C levels than cigarette smokers and lower HDL-C levels than non-smokers [85]. Traditional cigarette smokers switching to HTP have been shown to have reduced inflammatory markers, such as WBC count, and increased high-density lipoprotein levels [86].

In our study, uric acid, white blood cells, lymphocytes, and glucose levels were observed to be negatively correlated with the number of heated tobacco sticks consumed per day, suggesting a dose-dependent response. Moreover, the HDL level correlated positively with the daily number of heated tobacco sticks. This is confirmed by previous studies indicating that the risk of disease is dose-dependent and varies with the number of cigarettes smoked per day [87,88]. In another study, significantly lower plasma uric acid levels were noted in smokers than in non-smokers, and a significant negative correlation was noted with smoking status parameters, including the number of cigarettes smoked per day [89].

Low serum uric acid levels have been found to be associated with an increased incidence of lung cancer and chronic obstructive pulmonary disease (COPD) in cigarette smokers [90]. A low serum uric acid concentration is also associated with a higher incidence of cigarette smoking; in addition, current smokers with low serum uric acid levels have significantly higher rates of respiratory disease compared to smokers with elevated levels [90]. Previous research has found plasma uric acid concentrations to be lower in smokers than in non-smokers [89]. A study of workers in Japan found that people exclusively using heated tobacco products and dual users had significantly higher fasting glucose and HbA1c levels and a higher chance of developing prediabetes and diabetes than non-smokers [49].

The associations found for blood biomarkers in this study may still be influenced by uncontrolled confounders.

The morphology and biochemical parameters of younger people may be influenced by their lifestyle. As such, they should develop better lifestyle habits, such as eating a healthy balanced diet, engaging in physical activity, and refraining from smoking and consuming alcohol.

Blood tests are one of the basic diagnostic tools in medicine and can provide important information about health. However, the results can be affected by many factors, including diet and physical activity. Diets high in sugar can cause elevated glucose levels, uric acid, and altered platelet function [91].

Therefore, it is important for the patient to prepare properly for a blood test and to fast beforehand, especially in the case of glucose or lipid tests. To obtain the most reliable results, it is necessary to examine the products consumed by a patient and their lifestyle. A diet high in saturated fats can raise LDL levels, which can falsely suggest an elevated risk of cardiovascular disease, while eating a large amount of simple carbohydrates before the test can raise glucose levels, incorrectly indicating diabetes or pre-diabetes; in addition, intense physical exercise or stress can cause increased uric acid levels or glucose [92]. Numerous studies have shown that changing lifestyle improves blood parameters [93,94,95].

There is considerable interest in trying HTPs among non-smokers, and interest is very high among smokers [15]. A recent study conducted in Poland showed that 20.6% of adults surveyed had tried heated tobacco, and 10.9% declared that they had used it in the last 30 days, 4.9% of whom who used it every day [96]. Dual use of products, i.e., with traditional cigarettes or e-cigarettes, is also popular among young people [15]. One study found that every tenth adult (general population) studied was a dual user [96].

Smokers are particularly vulnerable to chronic diseases. It is important to note that all our surveyed IQOS users chose nicotine cartridges. The risk of developing chronic diseases among people using IQOS may be lower than among people who smoke traditional cigarettes.

One limitation of this study is that it uses only a single-point observation. In addition, the tested group was relatively small, and almost all the respondents were young adults and students; this selection may not be representative of the typical IQOS user, and as such, it may be difficult to extrapolate the results to older people, who may already be diagnosed with a chronic disease. Another limitation was that relatively few of the participants reported to the blood collection point for sampling: as a result, the blood data are only available for half the respondents. Furthermore, this study is based on self-assessment questionnaires, which were used to group the participants; as such, some errors may exist in their classification regarding HTP use, smoking traditional cigarettes, and not smoking. In addition, the results of the blood analysis may be influenced by differences in age, sex, and lifestyle factors, such as diet, physical activity, and alcohol consumption; these influences should be taken into account when interpreting the results. Few studies to date have measured blood parameters and compared them between smokers of both conventional cigarettes and heated tobacco products. The presented analyses (comparison of means, correlations) concern the relationship between the two key variables. While the authors are aware of the potential influence of other variables (i.e., confounding variables) due to the size of the examined sample (*n* = 195) and, in particular, the size of the subsample of people from whom biological material was collected (*n* = 110), it was not possible to conduct more complex analyses. This study nevertheless indicates potential directions for further research that would allow for verification of these issues.

The widespread use of HTP presents a challenge for public health. As such, it is important to develop effective educational policies and activities aimed at young people, who are emerging as the predominant market for these devices. Despite its limitations, this study has yielded interesting results that encourage further research. A broader set of laboratory test results may indicate the initial changes occurring in the bodies of young people using tobacco and nicotine products, and in a more long-term context, they could predict the incidence of chronic diseases.

## 5. Conclusions

No differences in blood counts, biochemical biomarkers, or blood lipid profiles were noted between IQOS users, traditional smokers, and non-smokers. Hence, heated tobacco products may only demonstrate a slightly lower risk compared to combustible cigarettes. Only a significantly higher platelet count was found in the IQOS users compared to the other groups. However, smoking and using HTPs can both cause short-term and long-term changes in blood morphology and biochemistry, which can increase the risk of various chronic diseases. Further cohort studies assessing the health status of young users of heated nicotine products and prospective analyses are necessary. Further longitudinal studies are needed, perhaps with a follow-up in a few years, to better understand the long-term effects of using heated tobacco products.

## Figures and Tables

**Table 1:** Characteristics of the study participants.

Variable	IQOS Smokers	Non-Smokers	Daily Smokers
	*n* = 65 (%)	*n* = 65 (%)	*n* = 65 (%)
**Gender**			
male	20 (30.8)	9 (13.8)	27 (41.5)
female	45 (69.2)	56 (86.2)	38 (58.5)
**Age**			
mean ± SD	21.23 ± 2.02	20.95 ± 1.81	21.43 ± 2.43
min–max	18–30	19–24	18–30
**Marital status**			
single	42 (64.6)	54 (83.1)	46 (70.8)
married	1 (1.5)	1 (1.5)	3 (4.6)
informal, stable relationship	22 (33.9)	10 (15.4)	16 (24.6)
**Education**			
general or vocational secondary schools	24 (36.9)	27 (41.5)	29 (44.6)
post-secondary	36 (55.4)	34 (52.3)	26 (40.0)
higher	5 (7.7)	4 (6.2)	10 (15.4)
**Place of residence**			
city up to 200,000 inhabitants	14 (21.5)	15 (23.1)	22 (33.8)
city above 200,000 inhabitants	44 (67.7)	31 (47.7)	36 (55.4)
village	7 (10.8)	19 (29.2)	7 (10.8)
**Student**			
yes	59 (90.8)	65 (100)	50 (76.9)
no	6 (9.2)	-	15 (23.1)
**Year of study**			
I–III	51 (86.4)	64 (98.5)	45 (90.0)
IV–VI	8 (13.6)	1 (1.5)	5 (10.0)
**Field of study**			
medical	53 (89.8)	64 (98.5)	35 (70.0)
non-medical	6 (10.2)	1 (1.5)	15 (30.0)
**University**			
public	59 (100)	65 (100)	50 (100)

Legend: *n*—number of participants; %—percent; min–max—minimum–maximum; SD—standard deviation; I–III year of study—Bachelor’s degree; IV–VI year of study—Master’s degree.

**Table 2:** Subjective assessment of the health status of the respondents and anthropometric measurements.

Variable	IQOS Smokers	Non-Smokers	Daily Smokers
	*n* = 65 (%)	*n* = 65 (%)	*n* = 65 (%)
**Subjective assessment of health status**			
very good	11 (16.9)	21 (32.3)	15 (23.1)
good	48 (73.9)	30 (46.1)	33 (50.8)
neither good nor bad	5 (7.7)	12 (18.5)	14 (21.5)
unsatisfactory	1 (1.5)	2 (3.1)	3 (4.6)
**Health status** (compared to the health status of peers)			
better	7 (10.8)	13 (20.0)	13 (20.0)
comparable	55 (84.6)	44 (67.7)	39 (60.0)
worse	3 (4.6)	8 (12.3)	13 (20.0)
**Symptoms occurring in the last month**			
toothache	11 (16.9)	12 (18.5)	11 (16.9)
chest pain after exercise	8 (12.3)	10 (15.4)	15 (23.1)
back pain	43 (66.2)	47 (72.3)	37 (56.9)
joint pain	27 (41.5)	18 (27.7)	23 (35.4)
constipation	13 (20.0)	16 (24.6)	16 (24.6)
headache	47 (72.4)	54 (83.1)	46 (70.8)
insomnia	29 (44.6)	24 (36.9)	39 (60.0)
depression	26 (40.0)	19 (29.2)	27 (41.5)
swelling (swelling of the feet)	6 (9.2)	5 (7.7)	8 (12.3)
**BMI** (body mass index)			
normal	45 (69.2)	58 (89.2)	37 (56.9)
overweight	16 (24.6)	6 (9.3)	23 (35.4)
obese	4 (6.2)	1 (1.5)	5 (7.7)
**WHR** (waist hip ratio)			
normal	53 (81.5)	43 (66.2)	50 (76.9)
abdominal obesity	12 (18.5)	22 (33.8)	15 (23.1)
**Number of steps per day**			
<10,000	42 (64.6)	46 (70.8)	33 (50.8)
≥10,000	23 (35.4)	19 (29.2)	32 (49.2)

Legend: %—percent; *n*—number of respondents.

**Table 3:** Blood parameters in IQOS users, non-smokers, and daily smokers.

	Study Group		Mean	Standard Deviation (SD)	Median	Minimum (Min)	Maximum (Max)	*p*-Value
		*n* = 110						
RBC [×10^6^/μL]	IQOS	37	4.78	0.38	4.79	4.13	5.77	0.17 *
	NS	45	4.61	0.40	4.67	3.97	5.74	
	DS	28	4.65	0.48	4.71	3.41	5.58	
WBC [×10^3^/μL]	IQOS	37	5.96	1.46	5.38	3.51	9.88	0.49
	NS	45	5.93	1.37	5.82	3.43	11.02	
	DS	28	6.38	1.51	6.13	4.24	9.65	
Lymphocytes [×10^3^/μL]	IQOS	37	2.09	0.60	2.03	1.05	3.87	0.78
	NS	45	2.16	0.53	2.11	1.10	3.69	
	DS	28	2.12	0.50	2.09	1.27	3.77	
MONO [×10^3^/μL]	IQOS	37	0.49	0.12	0.49	0.28	0.65	0.25
	NS	45	0.49	0.13	0.50	0.29	0.83	
	DS	28	0.57	0.17	0.52	0.28	0.92	
PLT [×10^3^/μL]	IQOS	37	290.27	66.40	284	166	422	*p* < 0.05
	NS	45	256.33	59.74	249	162	411	
	DS	28	267.14	50.33	263.50	184	411	
HGB [g/dL]	IQOS	37	14.33	1.07	14.30	12.70	17	0.05
	NS	45	13.53	1.33	13.70	8.60	16.70	
	DS	28	13.90	1.60	14	10	16.50	
CRP [mg/L]	IQOS	37	1.52	2.93	0.70	0.10	17.60	0.49
	NS	45	1.32	1.87	0.80	0.20	11.50	
	DS	28	1.88	3.83	0.60	0.10	16.70	
Uric acid [mg/dL]	IQOS	37	4.77	1.28	4.52	2.71	8.66	*p* < 0.01
	NS	45	4.40	0.87	4.28	2.77	6.82	
	DS	28	5.22	1.14	5.21	2.84	7.66	
Fibrinogen [mg/dL]	IQOS	37	239.05	53.08	228	168	380	0.85
	NS	45	237.47	60.02	230	157	502	
	DS	28	232.78	57.86	226	140	426	
Triglycerides [mg/dL]	IQOS	37	86.35	35.89	73	46	182	0.82
	NS	45	84.07	40.73	76	36	187	
	DS	28	87.46	48.10	69.50	34	271	
LDL [mg/dL]	IQOS	37	95.03	29.17	91	47	161	0.53 *
	NS	45	88.53	29.55	87	40	158	
	DS	28	89.14	22.07	86.50	41	128	
HDL [mg/dL]	IQOS	37	61.24	13.89	62	37	93	0.48 *
	NS	45	64.80	14.61	63	38	99	
	DS	28	61.75	13.80	61	42	93	
Total cholesterol [mg/dL]	IQOS	37	173.49	30.75	169	120	265	0.78 *
	NS	45	170.16	34.29	170	111	241	
	DS	28	168.21	25.41	169	129	213	
Apo A1 [g/L]	IQOS	37	1.56	0.29	1.56	1.06	2.25	0.77 *
	NS	45	1.60	0.28	1.59	1.14	2.23	
	DS	28	1.59	0.23	1.56	1.21	2.13	
Apo B [g/L]	IQOS	37	0.82	0.21	0.80	0.47	1.39	0.70 *
	NS	45	0.78	0.21	0.76	0.44	1.26	
	DS	28	0.79	0.18	0.77	0.50	1.19	
Glucose [mg/dL]	IQOS	37	85.25	8.72	85.90	58.60	112	0.23
	NS	45	86.86	5.76	87	77	99	
	DS	28	84.4	5.56	85	73.40	94	

Legend: RBC—red blood cell count; WBC—white blood cell count; MONO—monocyte number; PLT—platelet count; HGB—hemoglobin concentration; CRP—C-reactive protein; LDL—low-density lipoprotein; HDL—high-density lipoprotein; apo A1—apolipoprotein A1; apo B—apolipoprotein B; μL—microliter; g—gram; mg—milligram; L—liter; dL—deciliter; IQOS—IQOS users; NS—non-smokers; DS—daily smokers; * ANOVA test; significant values *p* < 0.05, *p* < 0.01.

**Table 4:** Correlation between the levels of blood parameters and the daily frequency of using nicotine-heated tobacco sticks among IQOS users (*n* = 37).

	ρ (rho)	*p*-Value
RBC and daily frequency of using nicotine-heated tobacco sticks	−0.16	0.3497
WBC and daily frequency of using nicotine-heated tobacco sticks	−0.36	*p* < 0.05
Lymphocytes and daily frequency of using nicotine-heated tobacco sticks	−0.38	*p* < 0.05
MONO and daily frequency of using nicotine-heated tobacco sticks	−0.23	0.1620
PLT and daily frequency of using nicotine-heated tobacco sticks	−0.17	0.3228
HGB and daily frequency of using nicotine-heated tobacco sticks	−0.26	0.1193
CRP and daily frequency of using nicotine-heated tobacco sticks	−0.13	0.4520
Uric acid and daily frequency of using nicotine-heated tobacco sticks	−0.34	*p* < 0.05
Fibrinogen and daily frequency of using nicotine-heated tobacco sticks	0.08	0.6200
Triglycerides and daily frequency of using nicotine-heated tobacco sticks	−0.19	0.2613
LDL and daily frequency of using nicotine-heated tobacco sticks	−0.08	0.6171
HDL and daily frequency of using nicotine-heated tobacco sticks	0.39	*p* < 0.05
Total cholesterol and daily frequency of using nicotine-heated tobacco sticks	0.01	0.9509
Apo A1 and daily frequency of using nicotine-heated tobacco sticks	0.29	0.0798
Apo B and daily frequency of using nicotine-heated tobacco sticks	−0.11	0.5070
Glucose and daily frequency of using nicotine-heated tobacco sticks	−0.47	*p* < 0.01

Legend: red blood cell count—RBC; white blood cell count—WBC; monocyte number—MONO; platelet count—PLT; hemoglobin concentration—HGB; C-reactive protein—CRP; low-density lipoprotein—LDL; high-density lipoprotein—HDL; apolipoprotein A1—apo A1; apolipoprotein B—apo B; the Spearman rank correlation coefficient—ρ (rho); significant values (*p* < 0.05; *p* < 0.01).

## Data Availability

The datasets analyzed during the current study are available from the corresponding author on reasonable request.

## Supplementary Materials

The following supporting information can be downloaded at: https://www.mdpi.com/article/10.3390/jcm14082734/s1. Table S1: Reference values of tested blood parameters; Table S2: Characteristics of the IQOS users; Table S3: Results of laboratory tests in the analyzed groups; Table S4: Effect size for ANOVA for PLT; Table S5: Effect sizes for ANOVA for blood parameters; Table S6: Logistic regression model for PLT.
