# Supplementary material for: Evaluation of Morphology and Biochemical Parameters of Young Adults Using Heated Tobacco Products in Poland: A Case-Control Study
Source: J Clin Med. 2025 Apr 16;14(8):2734. doi: 10.3390/jcm14082734 (PMC12028128; doi:10.3390/jcm14082734)
Supplement: Supplementary file 1 [file jcm-14-02734-s001.zip › jcm-3506265-supplementary.pdf]

Supplementary material

Table S1. Reference values of tested blood parameters

Parameter	Reference value
RBC [×10^6/ μL]	3.8-5.4
WBC [×10^3/ μL]	4.1-10.9
lymphocytes [×10^3/ μL]	1.00-3.70
MONO [×10^3/ μL]	0.00-0.70
PLT [×10^3/ μL]	140-400
HGB [g/dL]	12.0-16.4
CRP [mg/l]	<0.5
uric acid [mg/dL]	3.80-7.10
fibrinogen [mg/dL]	200-393
triglycerides [mg/dL]	<150
LDL [mg/dL]	<115
HDL [mg/dL]	>45
total cholesterol [mg/dL]	<190
apo A1 [g/l]	1.08-2.25
apo B [g/l]	0.55-1.55
glucose [mg/dL]	70-99

Legend: RBC- red blood cell count; WBC- white blood cell count; MONO- monocyte number; PLT- platelet count; HGB- hemoglobin concentration; CRP- C-reactive protein; LDL- low-density lipoprotein; HDL- high-density lipoprotein; apo A1- apolipoprotein A1; apo B- apolipoprotein B; μL- microliter; g-gram; mg-milligram; l-liter; dl, dL- deciliter

Table S2. Characteristics of the IQOS users (n=65)

	IQOS users *n*=65(%)
**How often heated tobacco products are used during the day**	
5	24 (37.0)
6-10	22 (33.8)
above 10	19 (29.2)
**How long do you use heated tobacco products?**	
less than 1 year	8 (12.3)
for 1 year	14 (21.5)
for 2 years	20 (30.7)
for 3 years	13 (20.0)
for 4 years	4 (6.2)
for 5 years	2 (3.1)
above 5 years	4 (6.2)
**Does the heated tobacco product contain nicotine?**	
Yes	65 (100)
**Age of initiation of use of heated tobacco products**	
11-15 years old	4 (6.2)
16-18 years old	29 (44.6)
19-21 years old	27 (41.5)
22-24 years old	5 (7.7)
**Noticed health symptoms caused by using heated tobacco products**	
Yes	21 (32.3)
No	44 (67.7)

**Which of the following symptoms caused by using heated tobacco products did you notice?**	
dizziness	9 (13.8)
headaches	8 (12.3)
nausea	6 (9.2)
problems falling asleep	2 (3.1)
breathing difficulties	7 (10.8)
chest pain	6 (9.2)
cough	6 (9.2)
accumulation of phlegm	5 (7.7)

Table S3. Results of laboratory tests in the analyzed groups

Parameter	Range	IQOS smokers *n*=37(%)	Non-smokers *n*=45(%)	Daily smokers *n*=28(%)
RBC [×10^6/ μL]	3.8-5.4	34(91.9)	42(93.3)	27(96.4)
	>5.4	3(8.1)	3(6.7)	1(3.6)
WBC [×10^3/ μL]	<4.1	2(5.4)	3(6.7)	-
	4.1-10.9	35(94.6)	41(91.1)	28(100)
	>10.9	-	1(2.2)	-
lymphocytes [×10^3/ μL]	1.00-3.70	36(97.3)	45(100)	27(96.4)
	>3.70	1(2.7)	-	1(3.6)
MONO [×10^3/ μL]	0.00-0.70	37(100)	42(93.3)	20(71.4)
	>0.70	-	3(6.7)	8(28.6)
PLT [×10^3/ μL]	140-400	34(91.9)	44(97.8)	27(96.4)
	>400	3(8.1)	1(2.2)	1(3.6)
HGB [g/dL]	<12.00	-	4(8.9)	3(10.7)
	12.0-16.4	36(97.3)	40(88.9)	24(85.7)
	>16.4	1(2.7)	1(2.2)	1(3.6)
CRP [mg/l]	<0.5	12(32.4)	7(15.6)	6(21.4)
	≥0.5	25(67.6)	38(84.4)	22(78.6)
uric acid [mg/dL]	<3.80	8(21.6)	11(24.4)	2(7.1)
	3.80-7.10	27(73.0)	34(75.6)	24(85.8)
	>7.10	2(5.4)	-	2(7.1)
fibrinogen [mg/dL]	<200	10(27.0)	14(31.1)	6(21.4)
	200-393	27(73.0)	30(66.7)	21(75.0)
	>393	-	1(2.2)	1(3.6)
triglycerides [mg/dL]	<150	34(91.9)	40(88.9)	27(96.4)
	≥150	3(8.1)	5(11.1)	1(3.6)
LDL [mg/dL]	<115	31(83.8)	35(77.8)	24(85.8)
	≥115	6(16.2)	10(22.2)	4(14.2)
HDL [mg/dL]	≤45	5(13.5)	4(8.9)	4(14.2)
	>45	32(86.5)	41(91.1)	24(85.8)
total cholesterol [mg/dL]	<190	28(75.7)	32(71.1)	21(75.0)
	≥190	9(24.3)	13(28.9)	7(25.0)
apo A1 [g/l]	<1.08	1(2.7)	-	-
	1.08-2.25	36(97.3)	45(100)	28(100)
apo B [g/l]	<0.55	2(5.4)	8(17.8)	2(7.1)
	0.55-1.55	35(94.6)	37(82.2)	26(92.9)
glucose [mg/dL]	<70	1(2.7)	-	-

	70-99	34(91.9)	45(100)	28(100)
	≥100	2(5.4)	-	-

Legend: RBC- red blood cell count; WBC- white blood cell count; MONO- monocyte number; PLT- platelet count; HGB- hemoglobin concentration; CRP- C-reactive protein; LDL- low-density lipoprotein; HDL- high-density lipoprotein; apo A1- apolipoprotein A1; apo B- apolipoprotein B; μL- microliter; g-gram; mg-milligram; l-liter; dl, dL- deciliter

Table S4. Effect size for ANOVA for PLT

PLT	point estimate	95% CI	
		Lower limit	Upper limit
Eta-squared (η^2)	0.058	0.000	0.150

Legend: PLT- platelet count; Cl- Confidence Interval

Table S5. Effect sizes for ANOVA for blood parameters

	Eta-squared (η^2)	95% CI	
		Lower limit	Upper limit
RBC	0.033	0.000	0.110
LDL	0.012	0.000	0.066
HDL	0.014	0.000	0.072
Cholesterol	0.005	0.000	0.042
Apo A1	0.005	0.000	0.044
Apo B	0.007	0.000	0.052

Legend: RBC- red blood cell count; LDL- low-density lipoprotein; HDL- high-density lipoprotein; apo A1- apolipoprotein A1; apo B- apolipoprotein B; Cl- Confidence Interval.

Table S6. Logistic regression model for PLT (above 265)

Variables	Standard error	Wald	df	p
Sex M1=M	0.523	1.355	1	0.244
Smoking=IQOS	0.506	4.056	1	0.044
Smoking=DS	0.594	2.399	1	0.121
BMI ⩽ 25	22793.150	0.000	1	0.999
BMI 25.01-30.00	22793.150	0.000	1	0.999
Does he/she take at least 10,000 steps a day	0.449	0.397	1	0.529

Age 20-24	0.739	4.877	1	0.027
Age 25+	1.074	1.047	1	0.306
Constant	22793.150	0.000	1	0.999

Legend: PLT- platelet count; M- Male; DS- Daily Smokers; BMI- body mass index; df- degrees of freedom; p-value
